# Authors’ correction for Euro Surveill. 2016;21(26)

**DOI:** 10.2807/1560-7917.ES.2016.21.31.30305

**Published:** 2016-08-04

**Authors:** Eurosurveillance editorial team

**Affiliations:** 1 European Centre for Disease Prevention and Control, Stockholm, Sweden

In the article ‘Letter to the editor: *Escherichia coli* harbouring *mcr*-1 gene isolated from poultry not exposed to polymyxins in Brazil’ by Lentz et al., published on 30 June 2016, the acknowledgements section was updated to include the name of Laurent Poirel. This change was made on 03 August 2016 at the request of the authors.

